# Stranger-directed consolation-like behavior in mice in a test of social decision making

**DOI:** 10.1016/j.yhbeh.2025.105831

**Published:** 2025-09-30

**Authors:** Sarah A. Blumenthal, Henry W. Kietzman, Karinne E. Cobb, Shannon L. Gourley

**Affiliations:** a Emory University Graduate Training Program in Neuroscience, USA; b Yale University School of Medicine, Department of Psychiatry, USA; c Emory University School of Medicine, Departments of Psychiatry and Pediatrics, USA; d Emory National Primate Research Center, USA; e Children’s Healthcare of Atlanta, USA

**Keywords:** Allogrooming, Self-grooming, Stress, Consolation, Empathy, Isolation

## Abstract

In the past decade, investigations into the neurobiology of empathy have been propelled by evidence that rodents are capable of more complex and nuanced social behaviors than previously believed. Several teams have reported that rodents will direct allogrooming and other consolation-like behaviors towards distressed conspecifics, including in situations in which consolation-like behavior was not the explicit focus of a given study. As a case in point, we unexpectedly found in a test of decision making incentivized by social experience that mice display consolation-like allogrooming towards distressed strangers. This observation was somewhat surprising because consolation-like behavior in rodents is often believed to be reserved for familiar conspecifics. Here in this brief report, we reveal that the allogrooming and close social proximity with a distressed stranger that we previously reported was accompanied by elevated sniffing and autogrooming in close proximity to the conspecific – a social contagion-like behavior. Also, these behaviors were not obviously attributable to general hyper-activity. We then describe the conditions in which this constellation of stranger-directed consolation-related behavior was observed, should this information support new research concerning stranger-directed consolation-like behavior.

## 1. Introduction

Social experiences influence our choices. For example, one might favor a restaurant where one previously dined with a romantic partner, that prior social experience conferring value to the restaurant. This process is thought to reconsolidate social memories and strengthen social bonds (Kietzman and Gourley, 2023). To investigate the ability of social experiences to influence later decision-making behavior, we recently developed a new task, termed the social incentivization of future choice (SIFC) (Kietzman et al., 2022; Kietzman et al., 2024). Mice first learn to respond in operant conditioning chambers for two food reinforcers. Next, they are placed in a novel chamber with an unfamiliar stimulus mouse or novel object alongside one of the two foods. Most mice will later prefer the ‘social’ over ‘nonsocial’ food, the social experiences adding value to the reward. The task thus quantifies the manner by which social experiences incentive later choice behavior. It differs from tasks examining the social transmission of food preferences, which measure the transfer of conspecific-mediated safety signals (Galef, 2003).

In establishing the SIFC task, we tested mice under several conditions, including when the stimulus mouse has been exposed to footshock. Video recordings of the social interactions revealed elevated allogrooming towards shocked mice, which we reported but did not explore further because it was not the focus of the investigation (Kietzman et al., 2022). Still, this observation is notable because allogrooming towards distressed conspecifics can be indicative of empathy-like consoling (Burkett et al., 2016). Consolation-like behavior in rodents is generally thought to be elicited by familiarity with a conspecific (Gonzalez-Liencres et al., 2014; Li et al., 2014; Rogers-Carter et al., 2018; Kitamura et al., 2024). Nevertheless, mice and rats, like humans, demonstrate consolation-like behavior towards unfamiliar conspecifics under some circumstances (Zeng et al., 2021; Matsumoto et al., 2021; Gachomba et al., 2024). As a case in point, mice in our report were unfamiliar. Here, we revisit archived data concerning these mice, analyzing more behaviors and revealing more evidence of stranger-directed consoling-like behavior. We then describe the conditions in which stranger-directed consolation-like behavior was observed, hoping to support new investigations into this phenomenon.

## 2. Methods

### 2.1. Subjects

Female C57BL/6 mice aged 2–6 months were used to avoid conspecific-directed aggression between unfamiliar male mice. Estrous cycle was not tracked. Mice were bred from Jackson Labs stock, maintained on a 12h light cycle (0700 on). Mice were given ad libitum access to food and water until instrumental response training. Procedures were performed in accordance with National Institutes of Health Guidelines for the Care and Use of Laboratory Animals and Emory University IACUC.

### 2.2. Instrumental response training

Mice were singly housed and food restricted to ~90 % of free-feeding body weight. Mice were placed in Med-Associates operant conditioning chambers equipped with two nose pokes and a food magazine. Mice were trained daily across seven sessions to nose poke for two distinct food reinforcers (chocolate or grain pellet), with responses at each aperture reinforced at a fixed ratio 1 schedule. Thirty pellets were available for responses at each aperture, and sessions ended when 60 pellets were earned, or after 70 min. If after seven days mice did not acquire all 60 pellets within 70 min, additional training occurred until all reinforcers were acquired. Responses/min were determined for the last seven training sessions, confirming no systematic preferences for either aperture (Kietzman et al., 2022).

### 2.3. SIFC

**Fig. 1.**
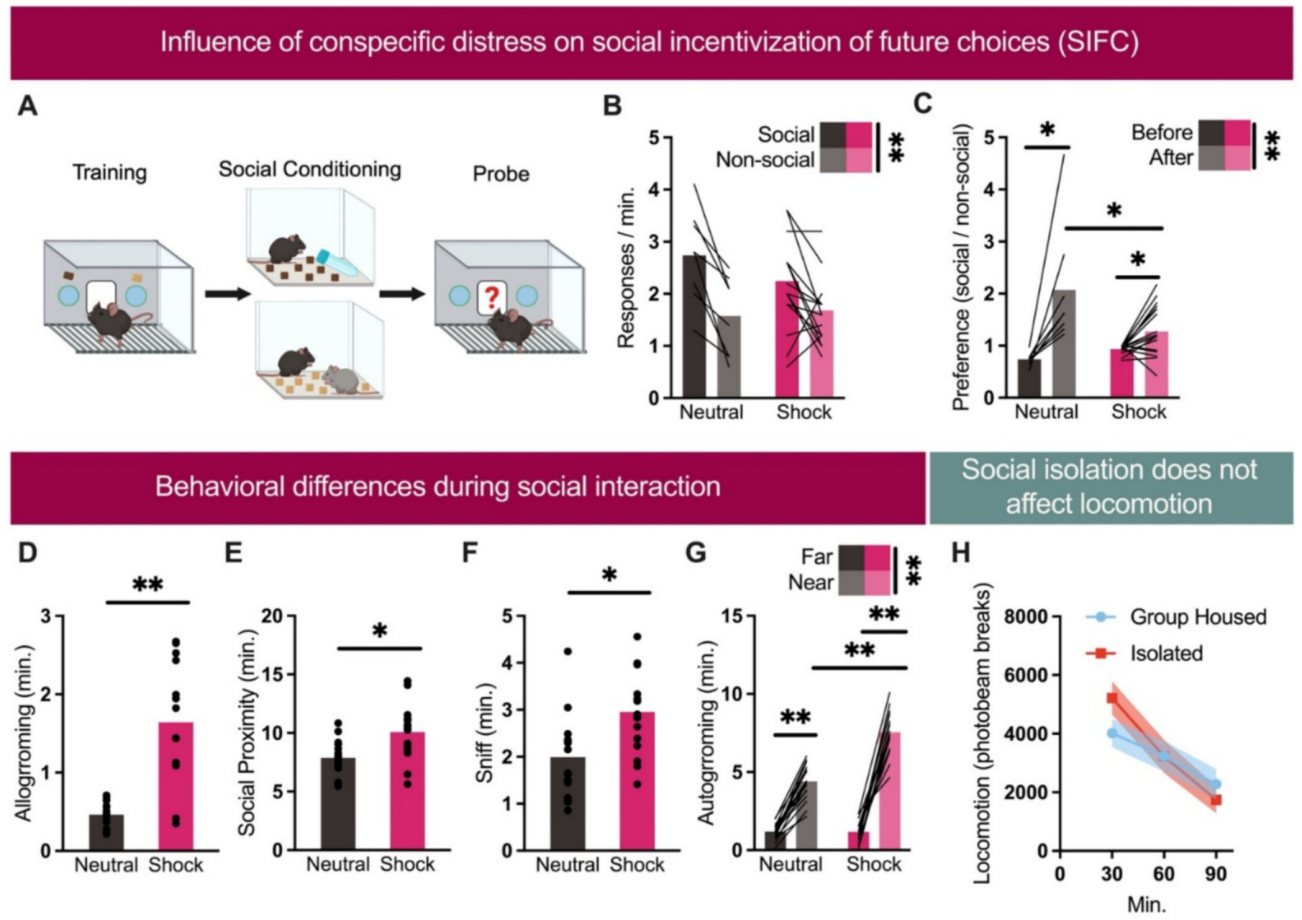
Influence of conspecific state on social incentivization of future choice (SIFC) and evidence of stranger-directed consoling behavior. a) SIFC procedure. Mice learn to respond for two distinct food reinforcers, which are later paired with a social interaction with an unfamiliar conspecific or an object as a nonsocial control. Later, responses at each port are recorded. Created in BioRender. Allen, A. (2025) https://BioRender.com/zgcgk3z. b) Mice respond more for the reinforcer paired with the social condition (main effect F(1,23) = 26.975, *p* < 0.001, η^2^ = 0.540; no effect of group F(1,23) = 0.405, *p* = 0.531, η^2^ = 0.017; no group x aperture interaction F(1,23) = 3.231, *p* = 0.085, η^2^ = 0.123). c) Interestingly, though, preference for the “social” reward is weaker following social conditioning with a shocked (distressed) demonstrator (main effect of testing phase F(1,23) = 28.945, p *<* 0.001, η^2^ = 0.557; no effect of group F(1,23) = 3.148, *p* = 0.089, η^2^ = 0.120; group x phase interaction (F(1,23) = 10.300, *p* = 0.004, η^2^ = 0.309). d) Exposure to a distressed conspecific during a social conditioning period increases durations of allogrooming (t(28) = 5.963, *p* < 0.0001, d = 2.182), e) time spent in social proximity (t(30) = 3.185, *p* = 0.003, d = 1.128), and f) sniffing (t(30) = 3.082, p = 0.004, d = 1.092). g) Autogrooming/eating is elevated when the experimental mouse is in close proximity with a shocked demonstrator, but not outside of a 1″ radius (main effect of group (F(1,31) = 36.943, p *<* 0.001, η^2^ = 0.544; main effect of proximity F(1,31) = 421.653, p *<* 0.001, η^2^ = 0.932; group x proximity interaction F (1,31) = 45.209, p *<* 0.001, η^2^ = 0.593). h) Separate mice were exposed to one week of isolation housing or maintained in groups, then they interacted with a novel conspecific as in the SIFC task, and locomotor activity was measured. Housing condition does not obviously impact locomotion (no main effect of group F *<* 1, η^2^ = 0.010; no group x time interaction F(2,24) = 2.427, *p* = 0.130, η^2^ = 0.310). A main effect of time (F(2,24) = 15.473, p *<* 0.001, η^2^ = 0.738) likely reflects habituation to the testing chamber. Bars and symbols reflect means+/−SEMs, and individual mice are represented. *p* values are * *<* 0.05, ** *<* 0.001.

Following training, mice underwent two conditioning sessions separated by 24 h and counterbalanced (Fig. 1a). In the “social” session, the experimental mouse was placed in a chamber (41×20×20 cm) with a novel age-matched conspecific and either chocolate or grain pellets on the floor. During the “nonsocial” session, mice were placed in a chamber with a novel object (15 mL falcon tube) and the other pellet on the floor. Sessions lasted for 60 min under low light. To bias against any potential pellet preference, we paired the “social” session with the pellet the mouse responded for less during training.

Mice were returned to the instrumental conditioning chambers for a 15 min probe test conducted in extinction. Response rates at each aperture were calculated, along with preference scores (social/non-social aperture). Preference scores were compared to pre-test preference scores, determined by dividing responses over the last seven days of training for the less preferred pellet (i.e., that later paired with the social interaction) over the more preferred one.

### 2.4. Footshock

The stimulus mouse above was either behaviorally naïve or subject to foot shock prior to social conditioning. Footshocks were administered at 0.5 mA for 2 s every 30 s for 5 min in a tubular foot shock chamber (5 × 1.5″; SD Labs). This protocol was adapted from (Sterley et al., 2018), which verified increased corticosteroid levels in both shocked and non-shocked partners.

### 2.5. Behavioral scoring

The first 30 min of the social conditioning session was scored by a single blinded observer. Time when the experimental mouse was allogrooming, in social proximity with the stimulus mouse, and sniffing the stimulus mouse were recorded. Social proximity was defined as a 1″ radius of the conspecific. Autogrooming and eating within a 1″ radius of the conspecific vs. outside of the radius was also scored because consolation-like behavior is accompanied by self-grooming, an “emotional contagion” response to a stressed conspecific (Burkett et al., 2016). Autogrooming and eating were combined due to inability to discern the two when mice faced away from the camera (Kietzman et al., 2022).

### 2.6. Locomotion

We ascertained whether the brief isolation that experimental mice experience prior to social conditioning impacts locomotion. Naïve mice were housed in isolation for 1 week or maintained in group housing. They were placed in a chamber with a novel conspecific and pellets, exactly as above. The conspecific mouse was removed, and locomotion was monitored by 16 infrared photobeams for 90 min.

### 2.7. Statistical analysis

Response rates, preference scores, photobeam breaks, and autogrooming/eating were compared using 2 × 2 repeated measure ANOVA, with Fisher’s LSD tests following interactions. Other behaviors were compared by unpaired *t*-tests. Some of the mice filmed for social behaviors were utilized in a separate experiment when a drug was administered during the probe test. Those probe test data are not reported here, which accounts for the difference in group sizes in Fig. 1. Further, 2 mice from the shock condition were not scored due to equipment failure, resulting in *n* = 8 neutral, *n* = 17 shock at top and n = 18 neutral, *n* = 15 shock at bottom of Fig. 1. The locomotion experiment used *n* = 6 social, n = 8 isolated mice. Values >2 standard deviations outside of the mean were considered outliers and excluded (total 3). *p* < 0.05 was considered significant, and data were analyzed by SPSS, with effect sizes calculated by Partial η^2^ and Cohen’s d.

## 3. Results

We examined how the state of an unfamiliar conspecific influences social and non-social behavior in mice by reanalyzing and extending data from our recent paper concerning how social experiences impact reward-related decisions (Kietzman et al., 2022). Mice first learned to nose-poke for two food pellets. One pellet was then paired with a novel conspecific, while the other was paired with an object. Half of the novel conspecifics had experienced footshock. After the social pairing, mice responded more for the pellet paired with the social experience (Fig. 1b) – reflecting the SIFC effect. We next calculated preference scores for the “social” vs. “non-social” aperture. Prior to conditioning, preferences were roughly 1 – i.e., no preference. After conditioning, this score increased as expected, but to a more moderate degree when the conspecific was shocked (Fig. 1c).

We next analyzed behaviors from the social conditioning phase, focusing on consolation-related behavior, as defined by (Burkett et al., 2016). Mice exposed to a shocked conspecific spent more time allogrooming (Fig. 1d) and in social proximity (Fig. 1e). These comparisons corroborate our prior report, which compared several different conditions in addition to these. Further, new analyses revealed more conspecific-directed sniffing (Fig. 1f). Autogrooming was also elevated when the experimental mouse was in close proximity to the shocked conspecific (Fig. 1g). This pattern is notable because rodents exhibiting consoling-like behavior also commonly increase autogrooming (Burkett et al., 2016), perhaps mirroring this stress-associated behavior in its shocked conspecific. The selective nature of this behavior here suggests that it relates to the conspecific and not non-specific hyper-activity.

One consideration is that experimental mice in the SIFC task are subject to social isolation, meant to elicit ‘social rebound’ later. An alternative possibility is that isolation instead simply elevates locomotion. We compared locomotion of mice that had been isolated for a week and then exposed to a novel conspecific, as in the SIFC task, vs. group-housed mice exposed to a novel conspecific. Isolation did not obviously trigger hyper- or hypo-activity (Fig. 1h).

## 4. Discussion

We find that mice tested in a socially-mediated decision-making task demonstrate allogrooming and other consolation-related behavior towards unfamiliar conspecifics that had been footshocked. These findings are notable, given that rodents often reserve consolation-like responses for familiar conspecifics (Rogers-Carter et al., 2018; Gonzalez-Liencres et al., 2014; Gachomba et al., 2024). Here we discuss the conditions in which we observed these behaviors, should this information be useful to those interested in studying stranger-directed consoling.

Our mice were part of now-published experiments determining how social experiences influence later reward-seeking behavior (Kietzman et al., 2022). Experimental mice were placed in isolation as part of the overall experimental protocol and thus entered into social interactions not having interacted with another mouse for at least a week. At this time, they demonstrated diverse consolation-related behaviors towards a distressed stranger, including elevated allogrooming, sniffing, and autogrooming, which could not obviously be attributed to hyper-activity. In contrast, multiple reports using socially housed rodents find no stranger-directed allogrooming (discussed (Yu et al., 2019)). A paper using group-housed female adult C57BL/6 mice, as here, that *did* report stranger-directed consolation-like behavior reported elevated allogrooming and sniffing but not social proximity towards the distressed conspecific (which we *did* observe) (Matsumoto et al., 2021). Possibly, a period of isolation potentiates or unveils consolation-like behavior towards strangers. Consistent with this notion, repeated experience with a stressed partner potentiates consolation-related behavior (Qu et al., 2023), and pain elicits stranger-directed allogrooming (Luo et al., 2020). If isolation similarly evokes consolation-like behavior towards strangers, this would be a useful tool by which to reliably induce and investigate this behavior. We speculate that isolation-evoked consolation may be restricted to isolation in adulthood, given that isolation in the juvenile age induces poor discrimination between conspecifics and objects and other atypical social behavior later in life (Bicks et al., 2020). Further, isolation in adolescence induces conspecific-directed darting, resembling social anxiety (Li et al., 2024).

A second factor may be that mice were females. That said, male rats and mice *can* display stranger-directed consolation-like behavior (Gachomba et al., 2024), so we think that sex is probably not a major factor. It could be a modulator, though, given that female rats appear more willing to approach a distressed conspecific (Rogers-Carter et al., 2018). Female mice are more prone to demonstrate socially transmitted fear from a stranger (Pisansky et al., 2017), and also prosocial responses to unconscious conspecifics (Sun et al., 2025). Potentially, oxytocin plays a role in any emergence of stranger-directed allogrooming, given that it facilitates consolation-like behavior (towards familiar conspecifics) in female mice (Matsumoto et al., 2021) and male voles (Horie et al., 2025).

We notably scored social interactions for 30 min, longer than often reported in the literature. It may be that organisms initially respond to a distressed stranger in an anxiogenic fashion, avoiding them (Rogers-Carter et al., 2018), then habituate, and ultimately engage in consolation-like behavior. Consistent with this possibility, one report that documented multiple consolation-like behaviors towards strangers used 30-min observation periods (Matsumoto et al., 2021). Meanwhile, other reports using 10-min observation periods documented merely ~10 s of stranger-directed allogrooming (Zeng et al., 2021; Kiyokawa et al., 2019).

Consoling familiar conspecifics is thought to recruit reward circuitry, yet the experience with a distressed stranger reduced preference for the food associated with the social interaction here. One implication is that consoling a stranger diminishes the rewarding nature of consoling; this observation could serve as a platform for understanding in- and out-group biases.

## Data Availability

Data will be archived and available through the Emory Dataverse.
